# P02.182. Integrative management of low back pain during pregnancy: a prospective case series

**DOI:** 10.1186/1472-6882-12-S1-P238

**Published:** 2012-06-12

**Authors:** K Pohlman, A Haan, C Long, T Rubley, K Roush

**Affiliations:** 1 Palmer College of Chiropractic, Davenport, USA; 2 Holzer Clinic, Gallipolis, USA

## Purpose

Fifty to 80% of pregnant women suffer from low back pain (LBP), a condition commonly treated by doctors of chiropractic (DC). Holzer Clinic, an Appalachian integrative health facility with 140 multispecialty physicians, utilizes both conventional prenatal and chiropractic care for their pregnant patients. This study assessed the feasibility to conduct a prospective case series of LBP patients receiving concurrent conventional prenatal and chiropractic care overseen by an off-site project manager (PM).

## Methods

Women age 21 and over receiving conventional prenatal care at Holzer Clinic who were referred to chiropractic care for LBP complaints were invited to participate in this IRB-approved study. The PM conducted a phone screen interview to determine eligibility. Completed baseline and 4 week questionnaires were mailed to the PM. Assessments included the Bournemouth Disability Questionnaire (BDQ), Numerical Rating Scale (NRS) for LBP, global improvement (GI) and adverse events (AE).

## Results

Thirty-nine women consented to participate in the study; 17 met all eligibility criteria. Exclusions were for age, LBP before pregnancy and no DC visit. Of the 13 participants who completed both questionnaires, all were white with a mean age of 28.4 (SD 1.1) and a median gestational age of 11.0 weeks (range 2-30). After 4 weeks of care the mean GI was 72%; mean improvement in BDQ was 5.8 (16%); and mean improvement in pain 2.3 (SD 2.18). AEs were mild (e.g. muscle soreness of less than 48 hours).

## Conclusion

Although patient outcomes were favorable in this small study, recruitment and follow-up data collection were challenging. It is important to investigate the effectiveness of integrative care for LBP during pregnancy; however, it will require the commitment of adequate resources and collaborations.

